# Rationale and methods for a randomized controlled trial of a movement-to-music video program for decreasing sedentary time among mother-child pairs

**DOI:** 10.1186/s12889-015-2347-4

**Published:** 2015-10-05

**Authors:** Pipsa P. A. Tuominen, Pauliina Husu, Jani Raitanen, Riitta M. Luoto

**Affiliations:** UKK Institute for Health Promotion Research, Tampere, Finland; Department of Health Sciences, Faculty of Sport and Health Sciences, University of Jyväskylä, Jyväskylä, Finland; School of Health Sciences, University of Tampere, Tampere, Finland

**Keywords:** Sedentary behavior, Physical activity, Movement-to-music, Motivational music, Video

## Abstract

**Background:**

Measured objectively, under a quarter of adults and fewer than half of preschool children meet the criteria set in the aerobic physical activity recommendations of the Centers for Disease Control and Prevention. Moreover, adults reportedly are sedentary (seated or lying down) for most of their waking hours. Importantly, greater amounts of sedentary time on parents’ part are associated with an increased risk of more sedentary time among their children. A randomized controlled trial targeting mother-child pairs has been designed, to examine whether a movement-to-music video program may be effective in reducing sedentary time and increasing physical activity in the home environment.

**Methods:**

Mother-child pairs (child age of 4–7 years) will be recruited from among NELLI lifestyle-modification study five-year follow-up cohort participants, encompassing 14 municipalities in Pirkanmaa region, Finland.

Accelerometer and exercise diary data are to be collected for intervention and control groups at the first, second and eighth week after the baseline measurements. Background factors, physical activity, screen time, motivation to exercise, and self-reported height and weight, along with quality of life, will be assessed via questionnaires. After the baseline and first week measurements, the participants of the intervention group will receive a movement-to-music video program designed to reduce sedentary time and increase physical activity. Intervention group mother-child pairs will be instructed to exercise every other day while watching the video program over the next seven weeks. Information on experiences of the use of the movement-to-music video program will be collected 8 weeks after baseline. Effects of the intervention will be analyzed in line with the intention-to-treat principle through comparison of the changes in the main outcomes between intervention and control group participants. The study has received ethics approval from the Pirkanmaa Ethics Committee in Human Sciences.

**Discussion:**

The study will yield information on the effectiveness of movement-to-music video exercise in reducing sedentary behavior. Intervention-based methods have proven effective in increasing physical activity in home environments. Music may improve exercise adherence, which creates a possibility of achieving long-term health benefits.

**Trial registration:**

The study is registered at ClinicalTrials.gov, as NCT02270138. It was registered on October 2, 2014.

## Background

The risk of many chronic diseases, among them type 2 diabetes, obesity, breast cancer, and cardiovascular diseases, along with all causes of mortality, grows in consequence of increasing of sedentary behavior (SB) and decreasing of physical activity (PA) [1, 2]. For our research, we have slightly adapted the definitions from the Sedentary Behavior Research Network [3] and Tremblay et al. [4] thus:

- Sedentary behavior is any waking behavior characterized by energy expenditure ≤1.5 METs while the participant is in a sitting or reclining posture. Screen time (watching television, using a computer, and playing video games or using other screens) is included in SB.
- Physical activity consists of meeting the established guidelines for physical activity [5], usually reflected in achievement of at least a certain threshold (see below) number of minutes of moderate to vigorous physical activity (MVPA) per day.
- Physical inactivity consist of performing insufficient amounts of MVPA – i.e., not following the specified physical activity guidelines.

The Centers for Disease Control and Prevention (CDC) recommends that adults engage in at least 150 min of moderate or 75 min of vigorous intensity activity, or an equivalent combination of aerobic activities every week in bouts of 10 or more minutes. In addition, muscle-strengthening activities for all major muscle groups on two or more days per week are recommended [5]. For children, the recommendation includes at least 60 min of aerobic activity per day and muscle and bone strengthening three or more days a week [5]. In addition, there are guidelines for both adults and children to reduce SB by minimizing their sitting, screen time, and motorized transportation [6–10].

By objective measurements, under a quarter of adults [11] and fewer than half of preschool children meet the criteria set in the aerobic PA recommendations [12, 13]. Furthermore, adults seem to be sedentary (sitting or lying down) most of their waking hours [11]. In addition, larger amounts of sedentary time on the part of their parents (such as watching TV) are associated with increased risk of higher sedentary time for children [14, 15]. Lifestyle interventions (such as promoting PA) targeted at both parents and children, aimed at parents’ participation in sports, and addressing PA level, along with considering the availability of media in the home and being involved in organized activities, may generate reduction in sedentary time and a PA increase among children [16].

In the context of PA and exercise, the benefits of music have been studied mainly for therapeutic purposes and in research involving athletes and other habitual exercisers. According to these studies, listening to music can influence exercise intensity, perceived exertion, and general mood, and can help to extend workout duration [17, 18]. Only a few studies have examined the ergogenic effect of motivational music and video interventions in combination. As music does, video watching has potential to shift attention from internal stimuli to external cues [19], and motivational music with video strengthens the positive effects of audiovisual interventions [19, 20]. Increasing PA and decreasing SB represents an opportunity to prevent health problems caused by inactivity and bring long-term health benefits. Screen time is usually quite sedentary in nature, accordingly, the use of music and video together should be able to yield added benefit for increasing PA.

An experimental study entitled “Movement-to-music video program for decreasing sedentary time among mothers and children” (Moving Sound) has been prepared with the aim of reducing sedentariness by introducing motivating movement-to-music video programs for mother-child pairs. To our knowledge, changes in SB or PA have not been studied previously in relation to a movement-to-music video program in the home environment.

This article describes the rationale behind the home-based movement-to-music video program for decreasing sedentary time while increasing mother-child pairs’ PA and the methods employed.

## Methods

### Design and ethics issues

A randomized controlled trial (RCT) is being used, with a parallel design involving one intervention and one control group of mother-child pairs. Mothers and children in the intervention group will receive a movement-to-music DVD and be instructed to exercise with it every other day. The target is to reduce SB and increase PA, in other words, sit less and be more active.

In the study, SB and PA are objectively assessed by means of an accelerometer in the first, second and eighth week of the intervention period. The accelerometers are attached to a flexible belt, which all women and children will be instructed to wear around their hips. The instructions specify using the accelerometers for 14 consecutive days (two weeks) during the participants’ waking hours at the beginning of the study and for seven days (one week) over the last week of the intervention. The accelerometers are to be removed before the participants go to bed and for showers, bathing, swimming, and other water activities. Measurements from accelerometers have been used in earlier studies for both adults and children, and they have been found to be a safe and reliable way to show changes in SB and PA [21]. There are no physical risks with the measurements in this study.

The information on the mothers’ background, motivation, and current health behavior comes from questionnaire data. The questionnaires are identical to those used in the Lifestyle, counseling, and exercise in maternity care (NELLI) five-year follow-up project.

Use of the movement-to-music video program as instructed (10–30 min every other day) will amount to less than half of adults’ and about 10–15 % of children’s PA guideline totals. The reason for this is to reduce risk of musculoskeletal injury and other complications. Moreover, when the movement-to-music video program is used in line with the instruction, there is no significant overall physical overload risk either. It is also assumed that adherence to the video protocol will be better with a short bouts of exercise compared to longer, although the evidence is contradictory [22].

Participation in the research is completely voluntary. Recruited mothers will give written informed consent for participation in the study on their and their child’s behalf. The persons recruited have the right to refuse to participate or to withdraw at any time from the study without explaining the reason.

This research project will be carried out in accordance with good scientific practice, with respect to ethics issues also. The UKK Institute for Health Promotion Research, based in Tampere, Finland, owns the data and is responsible for its storage and use.

The Pirkanmaa Ethics Committee in Human Sciences has issued a favorable statement on the Moving Sound study (statement 23/2014).

### Inclusion and exclusion criteria

Mothers and children are eligible for inclusion if they meet the following criteria: child included in the original NELLI cohort, child age of 4–7 years, the possibility of using a DVD player or the content behind a YouTube-link, and ability to perform PA and use the accelerometer as instructed. Mothers and children who because of medical factors (for example, chronic diseases, musculoskeletal or bone disorders, need for special rehabilitation, or trauma) are unable to perform PA will be excluded. Eligibility and willingness to participate in the research will be assessed via interviews of the participants during the personal contact of sampling for the NELLI study.

### Recruitment of the study population and randomization

Participants (i.e., mother-child pairs) in the Moving Sound study are being recruited from the cohort of the NELLI five-year follow-up study, which is based on the original cohort from the NELLI study (*n* = 837). The focuses of the NELLI work have been prevention of type 2 diabetes, metabolic syndrome, and obesity. The protocol and methods of the original NELLI study have been reported upon in detail previously [23].

**Fig. 1:**
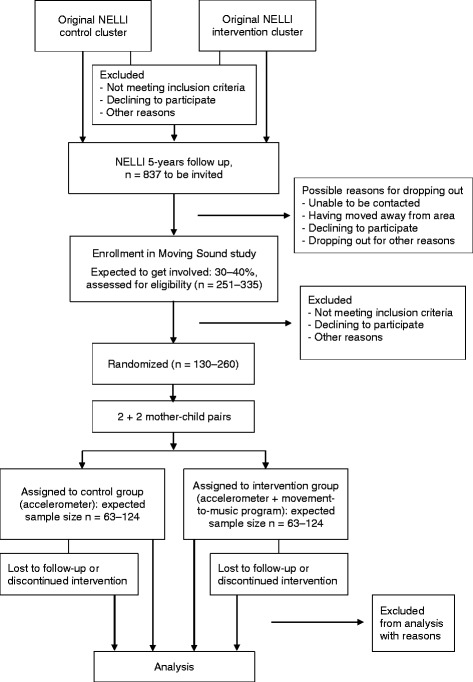
Flow chart, estimates for sample size, and randomization for the study

All NELLI follow-up study participants will be offered an opportunity to be part of the Moving Sound study. Mother-child pairs are being invited from 14 municipalities in the Pirkanmaa area, in south-western Finland, via an information letter and phone call. We expect to recruit 30–40 % of the original NELLI cohort (again, *n* = 837), for 251–335 mother-child pairs, in all. This expectation is based on figures from the original NELLI study, wherein 640 women agreed to participate and 399 women completed the intervention [24, 25]. Possible reasons for dropping out at this stage could include participants being unable to be reached or unwilling to take part in the study. Estimate of sample size and randomization for the study are shown in Fig. 1.

For the randomization, there was not an appointment order list and the total number of participants was unknown. Randomization was performed for blocks of four participants, in a 2:2 ratio: two mother-child pairs in the intervention group for two pairs of control participants. In practical terms, four random numbers were generated, and the pairs associated with the two largest were assigned to the experimental group and the two lowest to the control group. For allocation of participants, an appointment order list for the NELLI five-year follow-up study is being used. Mothers are randomized to the intervention or control group by means of sealed envelopes in the contact for sampling for the NELLI study.

The recruitment started in November 2014 and will continue until December 2015. Because season-to-season changes in Finland are large (with cold and snowy winters, rain in the spring and fall, and a warm summer), seasonal differences in PA levels may present themselves [26, 27]. Though the intervention takes place in the home, we have paid attention to this effect by performing the intervention throughout the year.

### Power calculations

On the basis of the Moving Sound pilot study, it is assumed that the mean sedentary (i.e., sitting or lying down) time will be 7 h 40 min per day at baseline. It is also assumed that the reduction in sedentary time in the intervention group at the end of the study will be around 6 % while the control group’s figure remains unchanged. Differences of groupwise means are tested via T-tests. Power calculations for the study (see Table 1) show that if the significance level is 0.05 (α = 0.05) and the power of the study is to be 80 % (β = 0.80), effect size varies from 0.357 to 0.500 and approximately 63–124 mother-child pairs per group are needed.

**Table 1:** Power calculations for the primary outcome of the study

Primary aim: decrease participants’ sedentariness by using movement-to-music video material							
Sedentary time (mean 7 h 40 min, α = 0.05)			Intervention: Accelerometer and DVD group		Control: Accelerometer group		Number of participants (mother + child pair) needed per group
Reduction of sedentary time	power	effect size	Mean	SD	Mean	SD	
5.4 %	β = 0.80	0.357	435 min	70 min	460 min	70 min	124
5.4 %	β = 0.80	0.385	435 min	65 min	460 min	65 min	107
5.4 %	β = 0.80	0.417	435 min	60 min	460 min	60 min	91
6.5 %	β = 0.80	0.429	430 min	70 min	460 min	70 min	86
6.5 %	β = 0.80	0.462	430 min	65 min	460 min	65 min	74
6.5 %	β = 0.80	0.500	430 min	60 min	460 min	60 min	63

α = significance level, β = power of the test

### The interventions

**Fig. 2:**
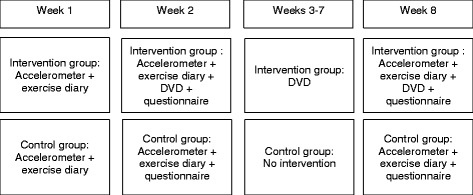
Intervention for eight weeks

The mothers and children will wear the accelerometer on an elastic belt on their hips for the first 14 days (two weeks) and last seven days (during week 8; see Fig. 2). Both groups will complete an exercise diary during weeks 1, 2, and 8. The intervention group will fill in questionnaires at baseline and after two and eight weeks, with the control group doing so at baseline and after the full eight weeks.

In addition, the intervention (i.e., movement-to-music) group will receive the DVD and YouTube-link to videos by e-mail or cell phone, in line with their choice between these, at the end of the first week. The DVD is the preferred option, because of the larger image and better sound when it is played through a television set. The YouTube-link is intended as an alternative to be used, for example, during trips or if the DVD breaks. Mothers in the movement-to-music group will be instructed to watch the program on the DVD or on the Internet every other day with their 4–7-year-old child. The mothers and children will be instructed to move to the video and report their exercise in the structured diaries. Mothers will also use the Brunel Music Rating Inventory-2 (BMRI-2) to assess the motivational quality of the music used in the movement-to-music video [28, 29].

## Outcome measurements and pre-testing

### Outcomes

**The main objective** of the study is to decrease SB and increase PA among the mothers and their children by means of the movement-to-music video program.

**The primary outcomes** of the study are SB and PA, which will be assessed objectively by means of the accelerometer and further examined via the exercise diaries and questionnaires. Measurements will be performed in the first, second and eighth weeks of the intervention. For inclusion in the analysis, accelerometer data for at least four days per week and measurement time of more than 10 h per day will be needed. Participants using the accelerometer on fewer than four days per week will be excluded. Any participants whose measurement time for a given day is over 20 h will be considered to have slept with the accelerometer. To avoid possible bias in SB time, the recording time for them will be capped at 20 h, with the deduction coming from their lying-down time. The measurement discriminates among the time spent in a sitting or reclining posture, standing still, and PA. The daily amount of standing-up (breaks in sedentary time) will be calculated from the number of lying/sitting periods that end with standing. Lying, sitting, and standing time, along with light, moderate, and vigorous PA time during waking hours, will be analyzed both in minutes and as a proportion of the measurement time (at least 10 h per day). In the analysis, moderate and vigorous activity might be combined as MVPA if vigorous PA covers a very small proportion of the total measurement time.

**Secondary outcomes** of the study include the quantity of self-reported sitting and screen time among mothers and children, motivation to exercise, and the motivational quality of the music and movement-to-music video. Additional secondary outcomes are mother’s weight and quality of life, depression, anxiety, perceived health, and work ability assessed by a questionnaire.

**The specific aim** is to study the effectiveness of the intervention by comparing accelerometer use alone with a combination of accelerometer and movement-to-music video program for mother-child pairs.

### Accelerometer measures and pre-test

The main aim with the project is to reduce sedentariness among mothers and their children. The accelerometers continuously measures tri-axial acceleration caused by any movement and permit precise assessment of individuals’ PA and SB both. Data on PA and SB will be collected in raw mode via a tri-axial accelerometer (Hookie AM 20, Traxmeet Ltd, of Espoo, Finland). The data will be analyzed as the mean signal amplitude deviation (MAD) of resultant acceleration for each epoch [21]. The choice of algorithms for use in the study is based on pilot studies conducted at the UKK Institute.

The resultant, which indicates the magnitude of the acceleration, is calculated for every measured sample. It is possible to determine with high accuracy whether the participant is standing, sitting, or lying down by applying the information from the three measurement axes of the accelerometer. Walking is used as a reference. While the body orientation during walking is upright and the direction of Earth’s gravity vector is constant, the vertical position (angle) of the accelerometer can be identified during normal walking. This known position (i.e., the angle of the accelerometer) can then be compared to other positions for purposes of recognizing different body postures. The number of instances of standing-up can be calculated from the number of lying/sitting periods ending with a clear vertical acceleration. In standardized conditions, standing can be distinguished from sitting or lying with 100 % accuracy, and sitting from lying with 95 % accuracy [Vähä-Ypyä et al., unpublished manuscript].

PA will be divided into three intensity categories by metabolic equivalent (MET): light, moderate, and vigorous. The classification was validated with simultaneous measurements of acceleration and oxygen consumption [30]. Light PA has been defined as activity corresponding to 1.5–2.9 METs, moderate activity as 3.0–5.9 METs and vigorous activity more than 6 METs [4, 5, 30].

Accelerometers were pre-tested with eleven 2–9-year-old children. The children engaged in free movement, play, and games on a test track field for one hour for determination of how scurrying-type movements appear in the readings. Another test, on a running track, tested speeds ranging from slow walking to participants’ maximum rate of running. In addition, accelerometers were piloted in free-living conditions with 10 children aged 4–7 for one week. In these tests, the acceleration signal behaved as expected, in other words, the MAD-value was higher for younger (smaller) children at the same speed and different activity intensities could be identified from the data.

### The movement-to-music DVD’s production and pre-testing

In spring 2014, three distinct movement-to-music video programs were prepared, by the Sibelius-Academy music-education students in course specifically on children’s music programs. The music was composed and arranged with lyrics. Further video programs were produced for the study specifically as part of a training course on children’s music and videos. Two of the videos last about 10 min each, including two songs and their movement instructions. There are three songs in all, because the title song, “Mutaveijarit” (or “The Mud Mates”) is part of both tracks (see Table 2). The third video includes all songs, with movements but without any verbal instructions, and it lasts about 12 min.

**Table 2:** Details of the music used for the movement-to-music DVD

Name of song	Music, lyrics, and arrangement	Music genre	Tempo (bpm^a^)	Motivational quality of the video vs. music group (BMRI-2^b^)
Video 1: Mutaveijarit ja karibialainen kala (10 minutes)				
”Mutaveijarit”	Eeva-Leena Pokela and Mutaveijarit	Children’s rock	94	34.6 vs. 33.7
“Karibialainen kala”	Aili Järvelä	Children’s Latin	128	34.0 vs. 31.9
Video 2: Kuraa ja mutaa (10 minutes)				
”Kuravelli”	Miia Reko and Mutaveijarit	Children’s folk	124	32.5 vs. 31.4
“Mutaveijarit”	Eeva-Leena Pokela and Mutaveijarit	Children’s rock	94	
Video 3: Mutaveijarit kooste (12 minutes)				
”Mutaveijarit”,”Karibialainen kala”,”Kuravelli”, and again”Mutaveijarit”				

^a^Beats per minute

^b^Brunel Music Rating Inventory-2 (max. 42 points)

To rate the motivational qualities of the three songs, a panel of eight physiotherapists (all female and comparable to the adult intervention participants in age, race, and cultural background) assessed each song by using the BMRI-2 [28, 29]. Another reason for pre-testing was to find out the influence of visual stimuli on the responses to the music. The BMRI-2 was translated into Finnish by investigators involved in the present study.

Each song was rated with a one number from 1 (“Strongly disagree”) and 7 (“Strongly agree”) for six statements about how much the characteristic features of the music would motivate a person during exercise. The range of total scores is 6–42, with scores below 24 indicating low motivational quality or an oudeterous (neutral) nature, those in the middle range (24–35) representing moderate motivation, and scores over 35 denoting highly motivating material [28].

The members of the video group (*n* = 4, mean age 41 years, SD 16.2 years) first watched the DVD and assessed all three songs separately, using the BMRI-2. Then they listened to the music only (without video) and rated the motivational quality of each song. The music group (*n* = 4, mean age 42.8 years, SD 15.8 years) assessed the motivational qualities of the songs first, then watched the DVD and appraised the music and video content together. In addition, both groups moved to the DVD and rated the motivational qualities of the music during movement. Music and video together received higher motivation ratings than did the music alone, from both groups (see Table 2).

### The movement program

The exercises in the videos are based on PA recommendations [5] and include exercises to improve or maintain aerobic fitness, muscle strength, balance, and coordination (including motor and rhythm coordination) [31]. All three songs begin with the Mud Mates getting up from a sofa. Each song has its own movements, which are performed to the beat of the music. The videos serve to encourage and motivate mother and child to exercise together and allow them to choose suitable movements for themselves from one to three variations.

The first song, “Mutaveijarit”, is accompanied by movements to improve aerobic fitness (walking, jumping, stepping, and shaking one’s whole body), postural balance (standing on one leg), and motor coordination (pelvic and midriff control, and agility). The second song, “Karibialainen kala”, involves movements to improve dynamic balance (moving the center of gravity to the edge of the area of support) and motor coordination (Caribbean dance movements such as swaying from side to side and making stepping motions). The third song, “Kuravelli”, entails movements to improve muscle strength (squats and lunges) and aerobic fitness (walking, jumping, and the side gallop). The last song, “Mutaveijarit”, (the same as the first) combines movement elements from all three previous songs.

### Data collection

The baseline data collection (with body-weight and questionnaire-based measurement) will take place during the personal contact of sampling for the NELLI study. Information on the accelerometer-based measurements will be supplied to participants at the same time. The timing of the data collection related to these measurements, the exercise diaries, and the questionnaires is described in detail in Table 3.

**Table 3:** Moving Sound data collection and measurements at baseline and the 1st, 2nd, and 8th week after baseline measurement

Data collection	Baseline	1st week	2nd week	8th week
Measurements and exercise diaries				
Accelerometer measurements		X	X	X
Exercise diaries		X	X	X
Weight	X			
Questionnaires				
Background questions	X			
Weight		X	X^a^	X
Earlier physical activity (LTPA)	X			
Current physical activity and sitting time	X		X^a^	X
Motivation to exercise (EIS and TPB)	X		X^a^	X
Quality of life (15 D)	X			
Depression (BDI)	X			
Anxiety (SAI)	X			
Work ability	X			
Perceived health	X			
Mother's musical background			X^a^	
Motivational quality of the music			X^a^	
Other motivation-related factors			X^a^	X^a^
Experiences of use of the video			X^a^	X^a^
Perceived changes in PA			X^a^	X^a^

^a^Intervention group

### The measurements and exercise diaries

Objective measurement of the SB and PA of the mothers and children will be conducted in the first, second and eighth weeks of the intervention via accelerometer use during waking hours. The mothers’ body weight will be measured at baseline.

Participants will be instructed to complete exercise diaries for the time for which they wear the accelerometers. The mothers will be asked to indicate their working hours and actual exercise, such as walking, jogging, running, swimming, biking, gym workouts, and dancing in the diaries. Start and end time of the exercise are to be filled in. Also, the participants will be instructed to assess the perceived exertion their exercise involves numerically: 1 = light PA, with no shortness of breath or sweating at all; 2 = moderate PA, with some shortness of breath or sweating; and 3 = vigorous PA, indicating heavy breathing or increased sweating. If more than one type of exercise is performed in the course of a day, the participants are to include all of these in the diaries. Mothers are asked to record the child’s exercise time at daycare or school and at home, and the time spent in PA, in the children’s diaries.

### Questionnaires

Information on participants’ background, PA, screen time, motivation to exercise, and self-reported height and weight, along with information on quality of life, will be collected at the baseline by means of the same questionnaires used for the NELLI five-year follow-up study (based on original and one-year follow-up questionnaires for the NELLI cohort). At two weeks after baseline, information on PA, screen time, self-reported weight, musical background, and motivation to exercise by means of a movement-to-music video program will be collected for the intervention group. At the eighth week after baseline, information on PA, screen time, motivation to exercise, and (self-reported) weight will be collected from all participants. Information on motivation to exercise by using the movement-to-music video program will be collected from the intervention group. Information on the children’s PA and screen time will be collected at baseline and the second and eighth week after baseline via questionnaires.

The information on participants’ background includes data on socioeconomic status, smoking, and height and weight. The participants of the intervention group will be asked for information on the mother’s musical background.

Mothers’ earlier PA will be examined at baseline via a leisure-time physical activity (LTPA) questionnaire, addressing the amount, duration, and intensity of PA within a typical week over the previous year. The validity and reliability of these questions have been examined previously [32]. The questionnaire on participants’ current PA and time spent in a sitting position in various contexts (on both weekdays and weekends) is the same as that utilized in the national Health 2011 Survey [33] and FINRISKI 2011 Study [34] in Finland, intended to ascertain how fully people meet the PA recommendations and how much they tend to sit. The questionnaire on the child’s typical exercise and screen time is based on the same questionnaire used in the Finnish project on health monitoring among children and young people (LATE) [35], with a protocol reported upon earlier [36]. There are separate questions on outside activities, exercises, and screen time. Both weekdays and weekends are covered. Responses to questionnaire items on current PA and on time spent in a sitting position and screen time will be examined at baseline and after week 8 for all participants, as will the figures for just after the second week for the intervention group.

The instrument examining motivation to exercise is based on the Finnish version [37] of the Enjoyment in Sport (EIS) questionnaire [38] and also addresses some factors motivating exercise [39]. Investigators involved in the present study modified the questions to be appropriate for exercising with children, for example, the statement “I like exercising” was changed to “I like exercising with a child.” Motivation and intention to encourage the child to perform PA will be examined by means of a short version of a theory of planned behavior (TPB) questionnaire [40]. In the first phase, this questionnaire was developed on the basis of the TPB manual instructions at University of Jyväskylä, Finland. In the second phase, it was pre-tested for clarity of language and suitability for the local culture by five experts in various relevant fields (physical education, exercise physiology, kinesiology, and health science). In the third phase, items with low reliability indices were excluded on the basis of pilot data collection from around 100 parents of 4–7-year-old children [41]. The final version of the TPB short form used in the present study is composed of elements on behavior, intention, attitudes to the behavior, and perceived control over the behavior. Motivation questions will be evaluated at baseline and after the eighth week for all participants and, in addition, for the intervention group after week 2.

The relationship between SB or PA and quality of life, depression, and anxiety is also of interest. Assessments of quality of life, depression, anxiety, and work ability will be performed via Finnish versions of the validated indicator. Quality of life will be assessed via the 15D instrument [42], depression via Beck’s Depression Inventory (BDI) [43], and anxiety by means of the State Anxiety Inventory (SAI) [44]. A visual analogue scale (VAS) will be used in the assessment of perceived health and an 11-point Likert scale for work ability. The 15D has 15 separate items: ability to be physically active, vision, hearing, breathing, sleeping, eating, communicating, elimination, normal functions, mental health, signs and symptoms, depression, anxiety, vitality, and sexuality. The BDI, in turn, is based on 21 distinct items for measuring the severity of depression in terms of a list of four statements. The SAI, a sub-scale of the State-Trait Anxiety Inventory (STAI), has 20 items for assessing “how I feel right now” on a four-point scale (“not at all” – “somewhat” – “moderately so” – “very much so”). Ten of the 20 statements typify presence and the other 10 absence of anxiety. Perceived health is an important factor when one wishes to predict functional capacity and health [45], and it will be measured via a VAS. Perceived work ability at the moment, relative to lifetime best, will be evaluated on an 11-point Likert scale, where 0 = total disability and, 10 = work ability at its best ever. Perceived deficiency of work ability in midlife is associated with accelerated weakening in health and functioning in later life [46]. Quality of life, depression, anxiety, perceived health, and work ability assessment will be evaluated for all participants at baseline.

In addition, there is a questionnaire for subgroup analysis, made up of questions for the intervention group. The items on mother’s musical background include questions about prior singing, playing of an instrument, dancing, and listening to music. The questionnaire on the motivational quality of the music is based on the BMRI-2. In this, the music will be rated on a scale of 1 (for strong disagreement) to 7 (for strong agreement) with each of six statements about how much the characteristic features of the music would motivate a person during exercise [29]. Experiences of use of the movement-to-music video program, factors (other than music) motivating exercise, and perceived changes in PA will be assessed via a questionnaire that was developed by investigators working on the present study. Participants will rate their experiences of use of the video on a scale of 1 (“Hard”) to 3 (“Easy”). Factors motivating exercise (the video’s characters, movements, and ambience and exercising with the child) will be rated on a scale of 1 (for “Strongly disagree”) to 7 (for “Strongly agree”). Perceived changes in PA will be rated between 1 (“Much less than earlier”) and 5 (“Much more than earlier”). In addition, participants will be asked about their actual activity while watching the video, including how well they performed the exercises in accordance with the video instructions, and about perceived personal shortcomings in physical fitness during completion of the video exercises. The questionnaires for subgroups were pre-tested by a group of eight physiotherapists and piloted in a separate pilot study. Items on mother’s musical background and BMRI-2 scores will be evaluated after second week, other questions after weeks 2 and 8.

### Statistical methods

The effects of the intervention will be analyzed in line with the intention-to-treat principle through comparison of the changes in the main outcomes between intervention and control group participants. Level of PA, sedentary time, and body weight will be compared between the intervention and control group. All differences in the afore-mentioned outcomes between groups will be examined via linear regression. If the assumptions of linear regression are violated, ordinal or logistic regression analysis will be used. Non-parametric methods (specifically, the Mann-Whitney *U*-test) will also be used to describe differences between groups if needed. Subgroup analysis within the intervention group will be conducted with the mother’s motivation to exercise with the movement-to-music video program and her musical background as covariates. The intervention group will be split into two classes on the basis of these (dummy) variables. These variables will be used to explain differences in exercise activity between mothers in the intervention group. A significance level of 0.05 will be used for all analysis.

## Discussion

The study under way is unique in executing an intervention for mothers and their children at the same time and focusing the intervention on reduction of SB and increasing of PA in the home environment. This is a new way to approach inactivity. The Moving Sound intervention has been designed for evaluation of the practical implementation of PA measurement and motivational music programs in combination. Given the well-known positive effects of music on mood, improvements are expected also with respect to exercise adherence [17]. For the intervention group, we have designed a pattern by which mothers and children can exercise together. This is promising, since PA interventions targeted at both adults and children have been shown to be highly effective in producing positive changes in sedentary time and levels of PA [16].

There are a few challenges in this trial. The first challenge involves the new data collection for the NELLI five-year follow-up study and Moving Sound sub-study. This has to do with response rate. Therefore, additional effort will be undertaken to increase participation, by such means as utilization of social media (a closed Facebook group), a small sports-related gift (such as a jump rope or Frisbee) for the child, and provision of feedback on the results. Another risk anticipated in the data collection involves the ability of hip-worn accelerometer to measure all movements during movement-to-music video use. The program features many shaking movements and jiggling of the hands and/or leg, and there might be individual differences on performance of these movements. Thus, it is unclear how all these kinds of movements can be detected by a hip-worn Hookie accelerometer. However, the accelerometer is able to detect overall PA and sedentary behavior [21] which is the main target of the study.

In conclusion, the use of music and video material together could be of added benefit for reducing SB and increasing PA for those mothers who have difficulties in exercising outside the home with young children. The intervention represents a possibility of achieving long-term health benefits by moving at home. Therefore, the study should show one way to improve activity level and thereby prevent otherwise forthcoming health problems.

## Abbreviations

BDI: Beck’s depression inventory
BMRI-2: Brunel music rating inventory-2
CDC: Centers for disease control and prevention
EIS: Enjoyment in Sport (a questionnaire)
LATE: The project for Finnish health monitoring among children and young people
LTPA: Leisure-time physical activity
MAD: Mean amplitude deviation
MET: Metabolic equivalent
Moving Sound: The movement-to-music video program study
MVPA: Moderate to vigorous physical activity
NELLI: A project on lifestyle, counseling, and exercise in maternity care
PA: Physical activity
RCT: Randomized controlled trial
SAI: State anxiety inventory
SB: Sedentary behavior
STAI: State-trait anxiety inventory
TPB: Theory of planned behavior
VAS: Visual analogue scale
